# Bunnell or cross-lock Bunnell suture for tendon repair? Defining the biomechanical role of suture pretension

**DOI:** 10.1186/s13018-015-0331-4

**Published:** 2015-12-29

**Authors:** Martin C. Jordan, Stefanie Hoelscher-Doht, Kai Fehske, Fabian Gilbert, Hendrik Jansen, Rainer H. Meffert

**Affiliations:** Department of Orthopaedic Trauma, Hand, Plastic and Reconstructive Surgery, University Hospital Wuerzburg, Oberduerrbacher Str. 6, 97080 Wuerzburg, Germany

**Keywords:** Achilles, Bunnell, Cross-lock, Pretension, Tendon, Suture

## Abstract

**Background:**

Suture pretension during tendon repair is supposed to increase the resistance to gap formation. However, its effects on the Bunnell suture technique are unknown. The purpose of this study was to determine the biomechanical effects of suture pretension on the Bunnell and cross-lock Bunnell techniques for tendon repair.

**Methods:**

Eighty porcine hindlimb tendons were randomly assigned to four different tendon repair groups: those repaired with or without suture pretension using either a simple Bunnell or cross-lock Bunnell technique. Pretension was applied as a 10 % shortening of the sutured tendon. After measuring the cross-sectional diameter at the repair site, static and cyclic biomechanical tests were conducted to evaluate the initial and 5-mm gap formation forces, elongation during cyclic loading, maximum tensile strength, and mode of failure. The suture failure mechanism was also separately assessed fluoroscopically in two tendons that were repaired with steel wire.

**Results:**

Suture pretension was accompanied by a 10 to 15 % increase in the tendon diameter at the repair site. Therefore, suture pretension with the Bunnell and cross-lock Bunnell repair techniques noticeably increased the resistance to initial gap formation and 5-mm gap formation. The tension-free cross-lock Bunnell repair demonstrated more resistance to initial and 5-mm gap formation, less elongation, and higher maximum tensile strength than the tension-free Bunnell repair technique. The only difference between the tensioned cross-lock Bunnell and tensioned Bunnell techniques was a larger resistance to 5-mm gap formation with the cross-lock Bunnell technique. Use of the simple instead of cross-lock suture configuration led to failure by suture cut out, as demonstrated fluoroscopically.

**Conclusion:**

Based on these results, suture pretension decreases gapping and elongation after tendon repair, and those effects are stronger when using a cross-lock, rather than a regular Bunnell suture. However, pretension causes an unfavorable increase in the tendon diameter at the repair site, which may adversely affect wound healing.

## Background

Achilles, patellar, and quadriceps tendon ruptures are common orthopedic injuries. Although surgical tendon repair is often indicated, the ideal suture technique remains unclear [1–6]. The most frequently described techniques are the Bunnell [7], Kessler [8], and Krackow sutures [9]. Regardless of the technique selected, the repaired tendon must be able to provide appropriate strength to resist loading during postoperative mobilization caused by physical therapy. This mobilization after tendon repair is important for preventing adhesions [10], which is one of the main causes of diminished range of motion after therapy. However, there is a significant risk of gap formation at the repair site during mobilization [11], which is a severe problem because gapping inhibits the development of strength and stiffness in the injured tendon that normally occurs during healing. Large gaps can even cause re-rupture during early rehabilitation [12].

Many different suture modifications have been described to improve the tendon repair strength and decrease gap formation. Increasing the number of suture strands crossing the repair site increases the resistance to failure [13], as expected. Including an epitendinous suture running circumferentially around the repair site not only adapts the frayed ends but also improves the biomechanical repair strength [14]. In addition, the suture diameter and material clearly affect the repair strength [15]. How the suture is anchored inside the parallel tendon filaments is known as the “locking configuration,” which is another major determinant of the repair strength and resistance to gap formation [16]. Increasing tendon purchase, that is the tendon area which is contained by the suture, also increases stability [17, 18]. However, limited attention was paid to suture pretension until recently [19, 20]. Tightening a suture should increase the resistance to gap formation, but that effect remains controversial [17, 20]. The effects of suture pretension on Bunnell sutures have not yet been reported, even though it is one of the most common techniques for tendon repair.

We hypothesized that pretensioning improves tendon repair strength and assessed both simple and a modified cross-lock Bunnell sutures because those repairs can be pretensioned. We biomechanically determined the initial gap force, 5-mm gap force, elongation, maximum tensile strength, and mode of failure to elucidate the benefits of suture pretension.

## Methods

### Specimens

We harvested 80 porcine hindlimb tendons. The tendons had a mean length of 122 ± 6.2 mm and a mean cross-sectional diameter of 46 ± 4.2 mm^2^, which is similar to the average size of a human Achilles tendon [21]. The cross-sectional diameter of each tendon was manually measured with calipers (area = π*ab,* where *a* is one half the tendon height and *b* is one half the tendon width). Tendons with obvious defects were excluded. After harvest and measurement, all of the tendons were wrapped in saline-soaked gauze and frozen at −20 °C. Prior to testing, the tendons were thawed for 12 h at 23 °C room temperature. The tendons were kept hydrated by a continuous saline spray to avoid desiccation. The reported testings were performed with approval of the local ethics committee.

### Suture technique

**Fig. 1:**
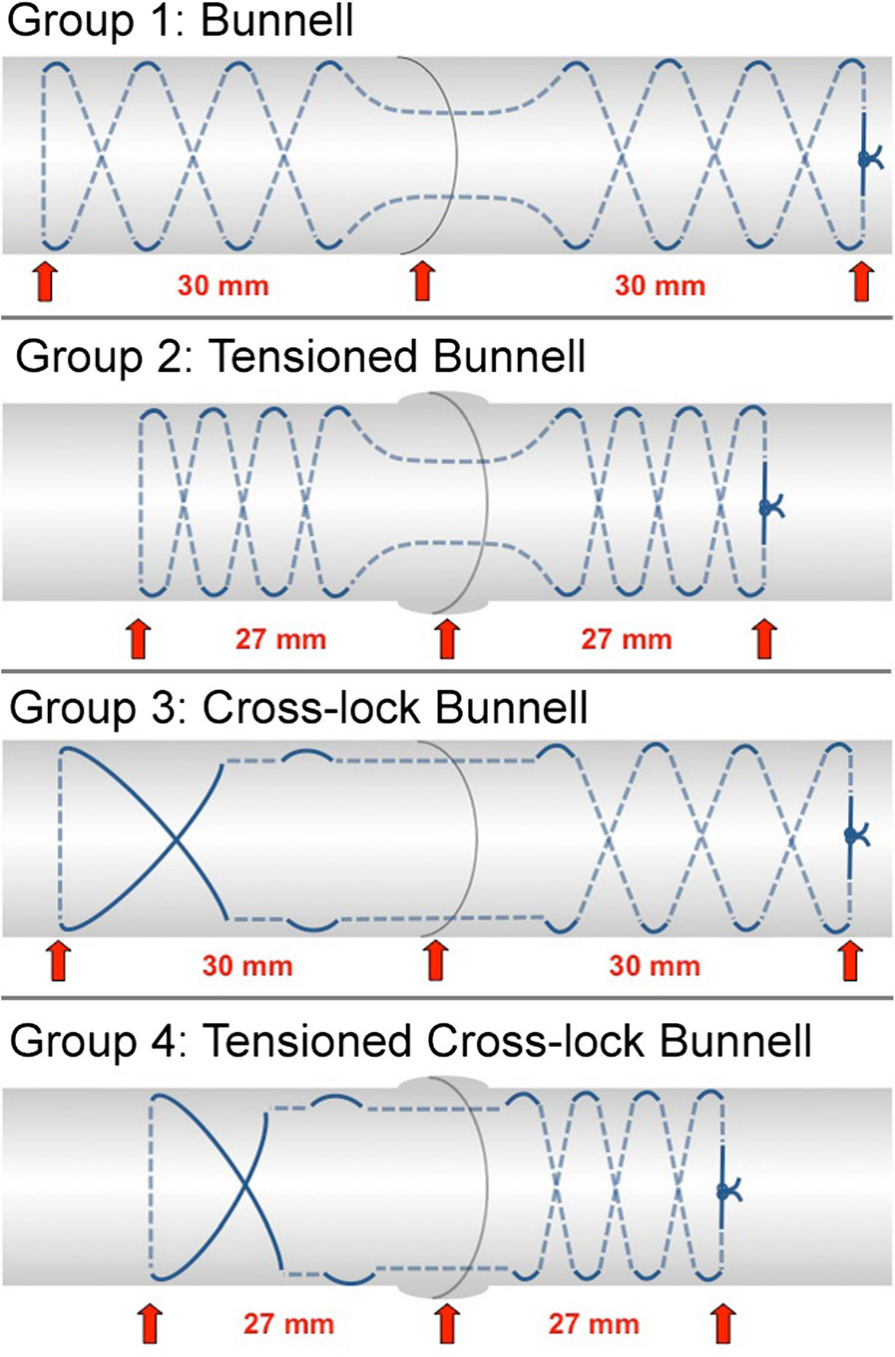
Illustration of the different groups tested. The tendon area encompassed by the core suture was shortened by 10 % on each side of the repair in groups 2 and 4 to perform suture tensioning

**Fig. 2:**
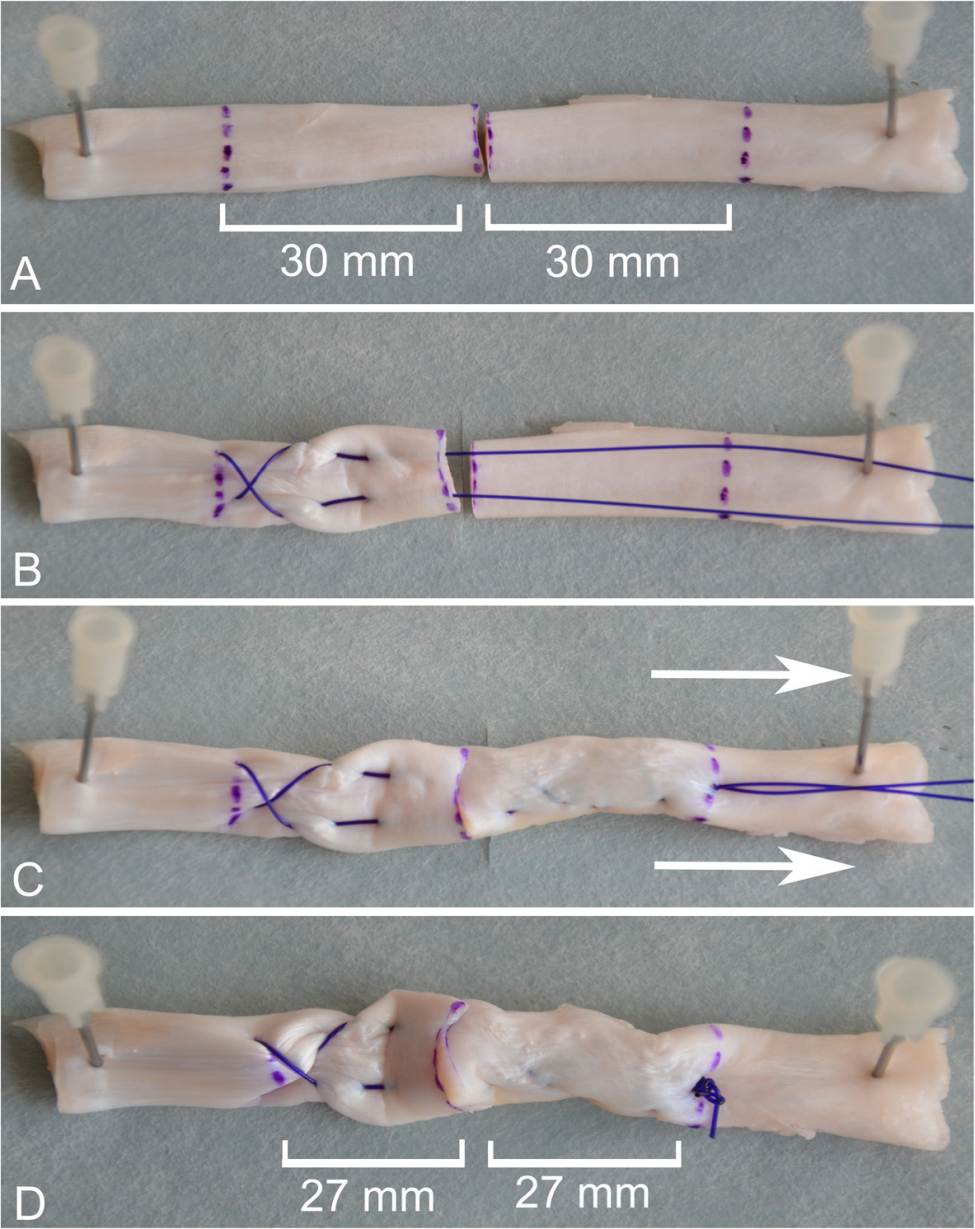
Additional steps for suture pretensioning. **a** Tendon purchase of 30 mm on each side of the defect. **b** Placing the distal part of the suture, including a cross-lock configuration. **c** The proximal part has a Bunnell configuration. Pretension was applied by pulling the suture in the longitudinal direction. **d** The tendon area encompassed by the core suture was shortened by 10 %

The tendons were randomly divided into four groups (Table 1, Fig. 1) and transected along their middle, leaving at least 50 mm on either side of the transection. Tendons in the Bunnell group (*n* = 20) were repaired using a two-strand Bunnell suture (PDS 1; Z631; Ethicon; Somerville; NJ, USA) and an additional epitendinous suture (PDS 5–0; Z3030; Ethicon). Tendons in the tensioned Bunnell group (*n* = 20) were repaired as in the Bunnell group, except here sufficient pretension to cause a 10 % shortening of the tendon segment encompassed by the core suture was applied at each side of the repair. The 10 % shortening was measured by marking the tendon purchase of 30 mm at the tendon stump before placing the suture. After placing the suture, pretension was applied by pulling the core suture in the longitudinal direction, thus shortening the tendon purchase down to 27 mm at each side of the transection (Fig. 2). Tendons in the cross-lock Bunnell group (*n* = 20) were repaired using a two-strand cross-lock Bunnell suture (PDS 1) and an additional epitendinous suture (PDS 5–0). Finally, tendons in the tensioned cross-lock Bunnell group (*n* = 20) were repaired with the cross-lock Bunnell suture, and pretension was applied as described above. The cross-sectional diameter of the tendon was measured before and after suture placement in each group to determine the increase in tendon diameter at the repair site. An epitendinous suture was included on all test specimens.

**Table 1:** Sample sizes and groups tested

Group	Technique	Specimens	Biomechanical testing
1	Bunnell	20	static (*n =* 10); cyclic (*n =* 10)
2	Tensioned Bunnell	20	static (*n =* 10); cyclic (*n =* 10)
3	Cross-lock Bunnell	20	static (*n =* 10); cyclic (*n =* 10)
4	Tensioned Cross-lock Bunnell	20	static (*n =* 10); cyclic (*n =* 10)

**Fig. 3:**
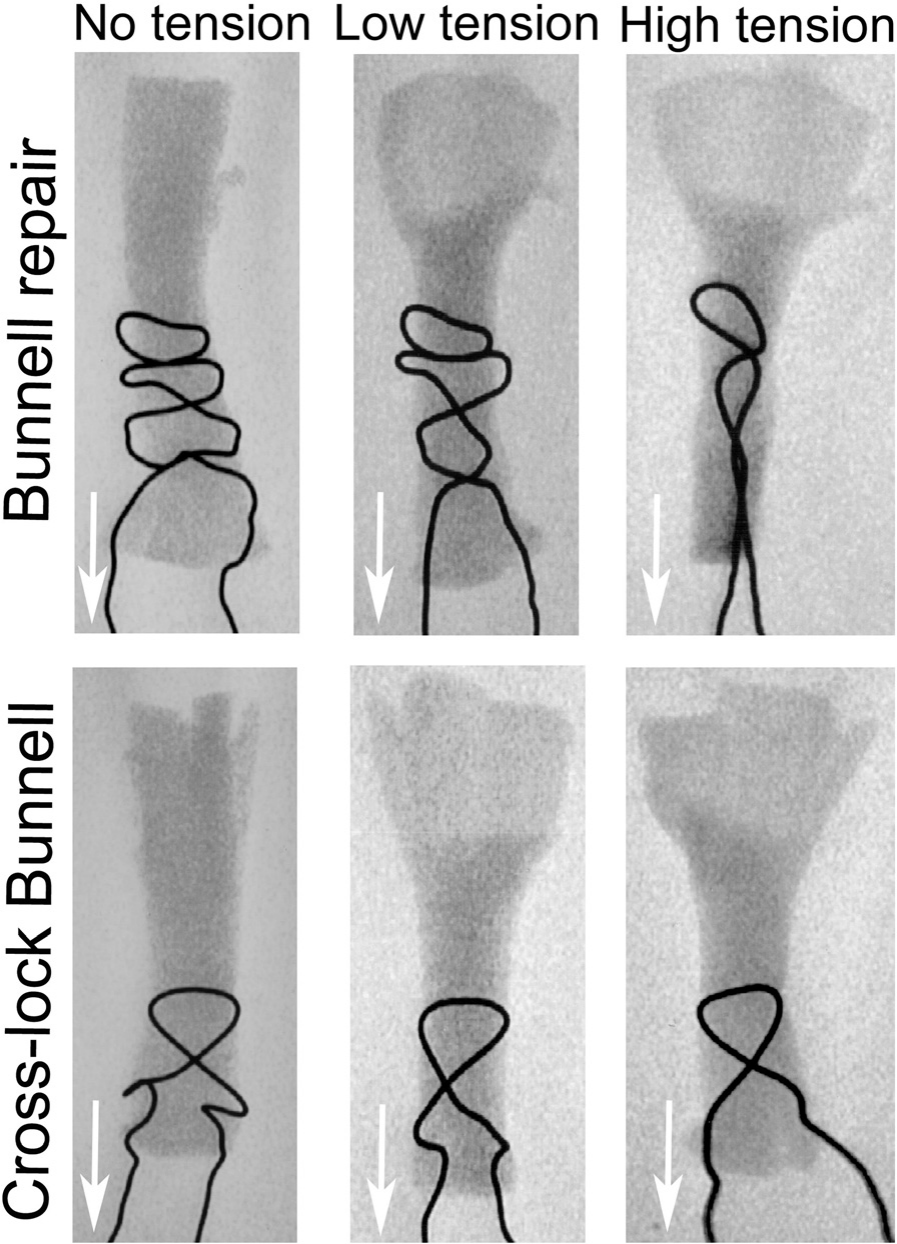
Fluoroscopic examination of the suture failure mechanism. Different tension loads were applied to the Bunnell and cross-lock techniques. Suture cut out occurred at lower tension levels in the Bunnell technique, which may explain the lower resistance to gap formation

### Biomechanical testing

Testing was conducted on a mechanical testing machine (Z020, Zwick/Roell GmbH; Ulm, Germany) using test Xpert II software (Version 3.3; Zwick/Roell). Uniaxial testing was performed with a 20-kN load cell and two stainless steel clamps. The distance between the two clamps was set as 80 mm and both ends of the tendon were clamped onto the machine. The tendon ends were rapidly frozen with liquid nitrogen for 60 s before being fixed into the clamps. Static (10 tendons per group) and cyclic testing (10 tendons per group) were performed (Table 1). The static, load to failure test included a preload of 3 N and a tendon distraction rate of 20 mm/min. The initial gap formation (loss of contact between the tendon ends) and 5-mm gap formation forces, ultimate tensile strength, and mode of failure were measured. The cyclic testing included a preload of 3 N and a preconditioning loading to 10 N for 10 cycles. Subsequently, cyclic loading between 20 and 100 N was applied for 100 cycles at a distraction rate of 20 mm/min. The load and displacement were continuously recorded to generate a load-displacement curve. The displacement curve for the 100 cycles was analyzed to determine the amount of elongation. We further evaluated the mechanism of suture failure on two other tendons. These tendons were repaired using a 1 mm stainless steel wire with the Bunnell or cross-lock Bunnell suture techniques. While under axial load, the mode of failure was determined by fluoroscopy (Fig. 3).

**Table 2:** Biomechanical results for the different groups

Technique	Initial gap force (N)	5-mm gap force (N)	Maximum tensile strength (N)	Elongation during cyclic load (mm)
Bunnell	61.3 ± 10.2	79.9 ± 12.3	145.5 ± 27.9	7.9 ± 3.4
Tensioned Bunnell	151 ± 20.5	128.5 ± 30.3	194 ± 11.8	4 ± 0.5
Cross-lock Bunnell	93.1 ± 15.4	116.9 ± 13.3	179.1 ± 23.7	5.6 ± 2.5
Tensioned Cross-lock Bunnell	149.9 ± 13.7	182.2 ± 22.7	192.8 ± 29.5	4.4 ± 1.5

**Fig. 4:**
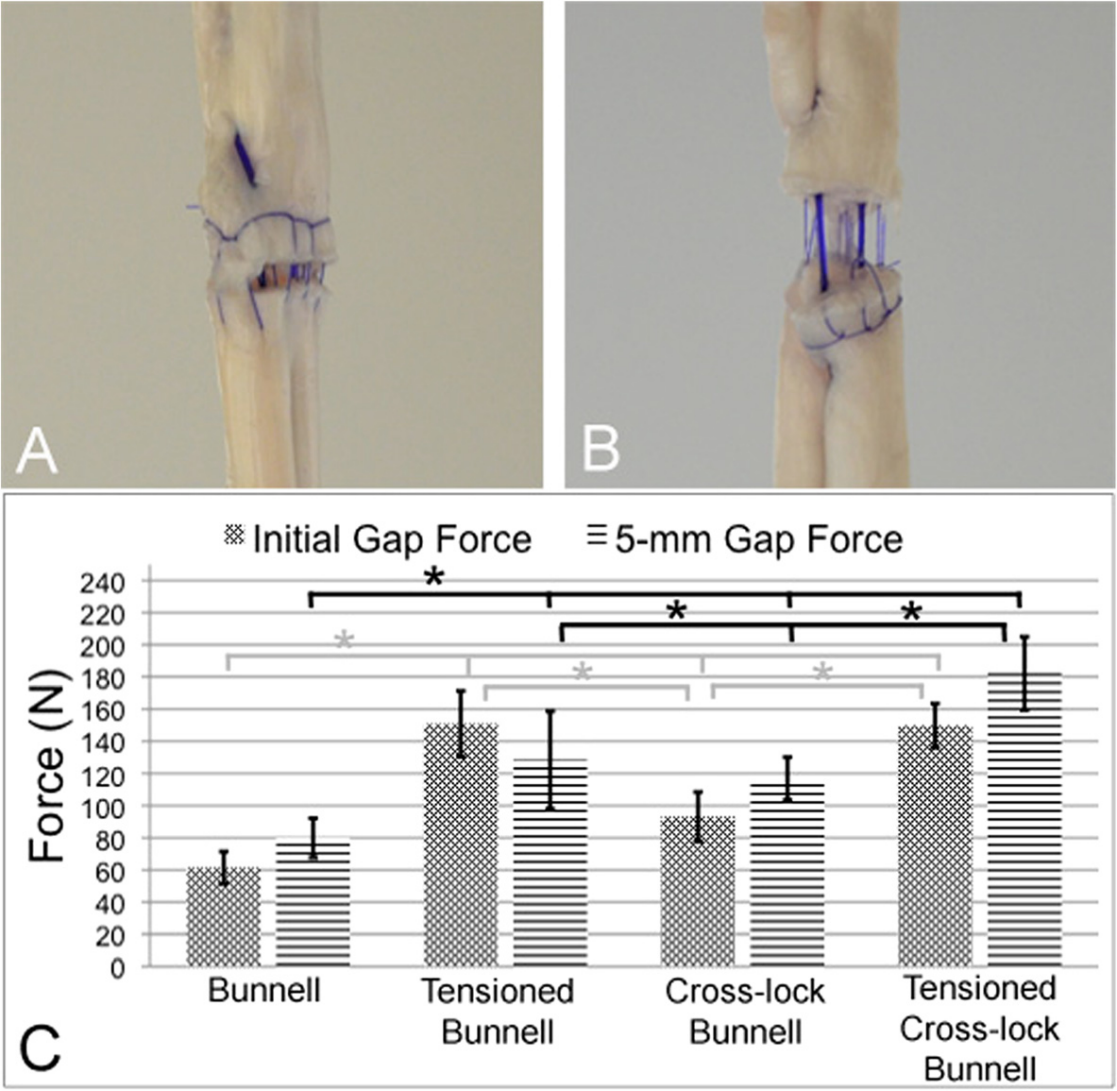
Gap formation. **a** Initial gap formation. **b** 5-mm gap formation. **c** Bunnell suture without pretension had less resistance to initial gap formation than all of the other repairs (*p* < 0.001). Also, significantly more load was needed to produce a 5-mm gap in the tensioned Bunnell (*p* < 0.001), cross-lock Bunnell (*p* < 0.002), and tensioned cross-lock Bunnell (*p* < 0.001) groups than in the simple Bunnell suture group. Pretension significantly increased the resistance to gap formation in the Bunnell (*p* < 0.001) and cross-lock Bunnell (*p* < 0.001) repairs

### Statistics

The results are shown as means with standard deviations. A power analysis was performed to show that the sample size was sufficient. Shapiro-Wilk tests were performed to assess the variable distributions. Thereafter, ANOVAs were calculated, and for significant effects, post hoc Tukey tests were used to compare group means (SPSS Inc.; Chicago; IL; USA). *P* values less than 0.05 were considered statistically significant.

## Results

### Initial gap force

The initial gapping appeared at the lowest tensile force in the simple Bunnell repair group. The cross-lock Bunnell repair without pretension showed a higher resistance to initial gap formation compared with the simple Bunnell repair (*p* < 0.001). Pretension increased the gap formation force by 146 % in the simple Bunnell (*p <* 0.001) and 61 % in the cross-lock Bunnell (*p* < 0.001) repairs (Table 2, Fig. 4).

### Five-millimeter gap force

The tension-free Bunnell suture showed the least resistance to 5-mm gap formation. Pretensioning increased the resistance by 60 % (*p* < 0.001) in the Bunnell and by 56 % in the cross-lock Bunnell (*p* < 0.001) repairs. In the tensioned Bunnell suture, the 5-mm gap force was lower than the initial gap force because the epitendinous suture ruptured before the 5-mm threshold. This caused a sudden decrease in the tensile force (Fig. 4).

### Maximum tensile strength

**Fig. 5:**
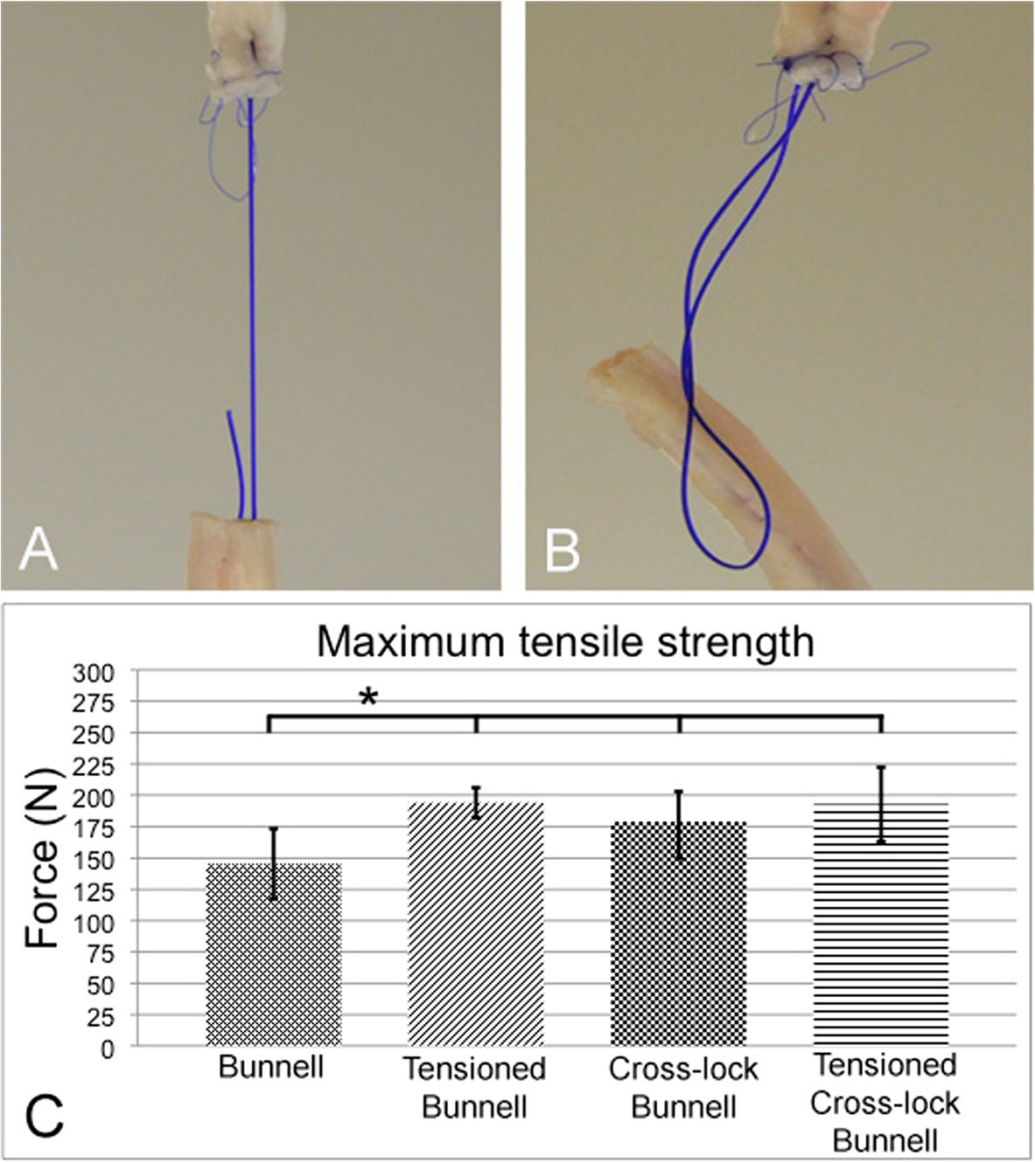
Maximum tensile strength. **a** Failure by suture rupture. **b** Failure by suture pullout. **c** Tension-free Bunnell repair showed a significantly lower maximum tensile strength than the tensioned Bunnell (*p* < 0.001), cross-lock Bunnell (*p* < 0.019), and tensioned Bunnell (*p* < 0.001)

The tension-free Bunnell suture had the lowest maximum tensile strength. However, it was significantly increased with pretensioning (*p* < 0.001). The maximum tensile strength was higher in the cross-lock Bunnell repair than in the simple Bunnell repair (*p* < 0.019) but was not significantly increased by tensioning (Fig. 5).

### Elongation

**Fig. 6:**
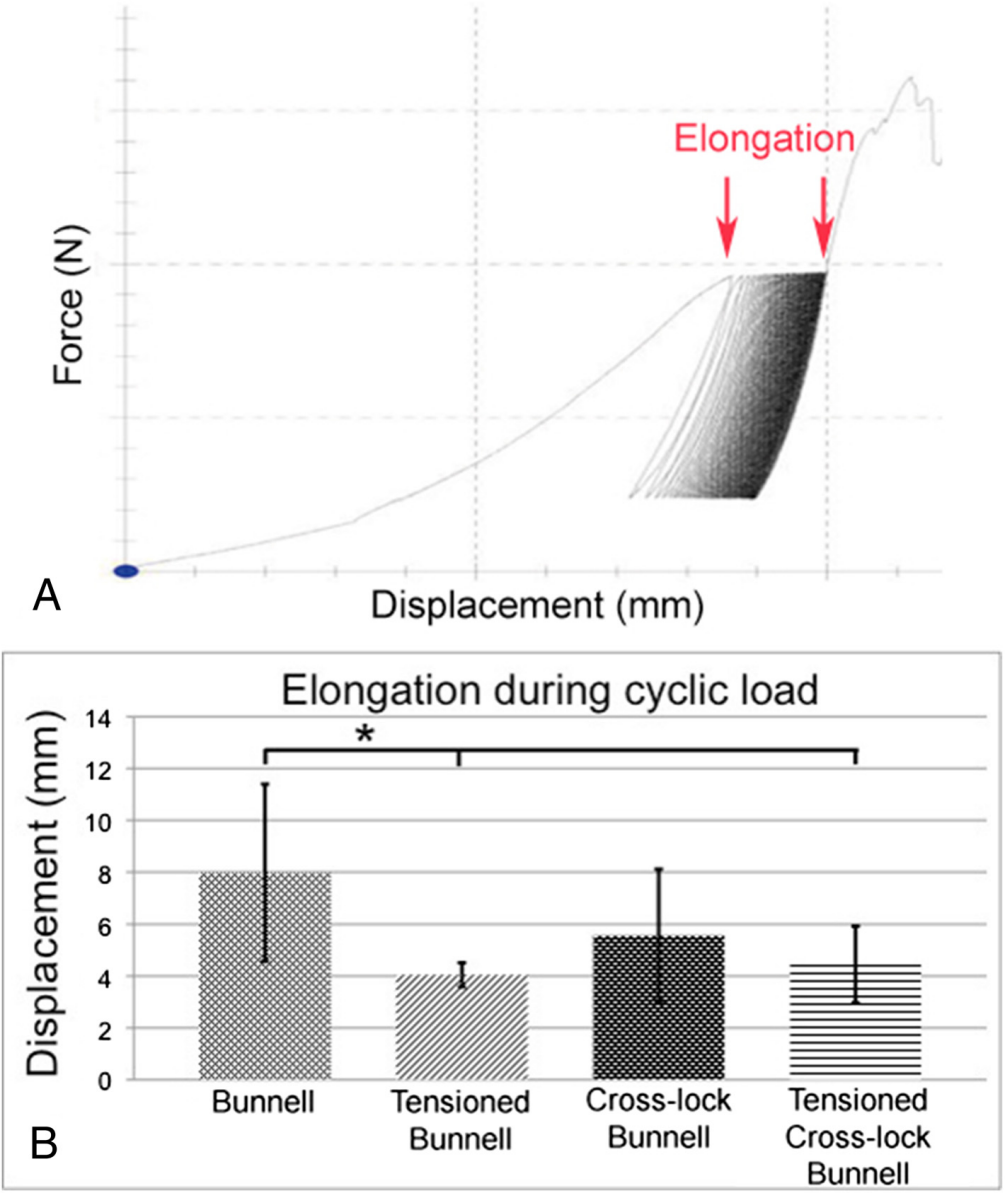
Elongation. **a** Amount of Elongation during cyclic load marked by red arrows. **b** During cyclic loading, the largest elongation occurred in the Bunnell repair without pretension, and it was significantly larger than that in the tensioned Bunnell (*p* < 0.002) and tensioned cross-lock Bunnell (*p* < 0.007) repairs

Elongation during cyclic testing indicates the lengthening of the repaired tendon. The Bunnell suture without pretensioning showed the highest elongation. Significantly less elongation was found in the tensioned Bunnell (*p* < 0.002) and tensioned cross-lock Bunnell (*p <* 0.007) repair (Fig. 6).

### Mode of failure

The mode of failure was determined under static conditions (*n* = 10). In the Bunnell and pretensioned Bunnell groups, failure occurred in 60 % of the tendons by suture pullout and in 40 % by suture rupture. The cross-lock Bunnell and tensioned cross-lock Bunnell repairs all failed by suture rupture. The mechanism of failure was observed under fluoroscopy in additional specimens (*n* = 2). Axial tension on a Bunnell type suture results in stringing followed by suture cut out, which promotes gap formation. No stringing or cut out was observed under similar loads in the cross-lock configuration (Fig. 3).

### Diameter increase

The average cross-sectional diameters after suture placement were 81.2 ± 6.1 mm^2^ in the Bunnell and 80.2 ± 12.9 mm^2^ in the cross-lock Bunnell groups. After shortening, the diameters were 89.2 ± 13.1 mm^2^ in the tensioned Bunnell and 92.9 ± 11.1 mm^2^ in the tensioned cross-lock Bunnell groups. Thus, pretensioning caused an increase in diameter at the repair site. The cross-sectional diameter increased by 10 % in the Bunnell and 15 % in the cross-lock Bunnell groups.

## Discussion

Gapping and lengthening of the tendon stumps are reported to impair proper healing of repaired tendons [12, 22–24]. Suturing of the tendon should ensure that the tendon ends remain attached and avoid the development of a gap at the repair site or lengthening, thereby allowing safe postoperative mobilization. The results reported here confirm that the application of suture pretension increases the resistance against gap formation and lengthening in the simple and cross-lock Bunnell sutures.

The application of suture pretension causes overlapping and stringing of the torn ends of the tendon, and more tensile strength will be required to pull the stumps apart. However, pretension can also cause several problems that have not yet been reported. First, suture tensioning increases the tendon diameter at the repair site, here by 10 to 15 % compared with the tension-free repairs. Even if this diameter increase does not affect the sliding properties, it is unclear what magnitude of increase is tolerable and what will increase the friction. Whenever possible, the peritendineum should be closed over the repair to encourage sliding, and the increase in tendon bulk should not compromise this coverage. Most importantly, the skin must be securely closed and any diameter increase should not put pressure on the overlying skin to ensure appropriate wound healing. This point applies to all anatomic locations, especially those with a limited amount of surrounding soft tissue. Second, tensioning shortens the overall tendon length, which could limit the final range of motion. Third, the use of pretension in high-strength locking repairs may be difficult. For example, the well-known Krackow repair [9] seems less suitable for the use of pretension because after the first locking loops are placed, no additional tension can be applied because the loops are closed and the repair is secured. Thus, it could be problematic to implement pretension in full locking repairs. This might also explain why Krushinski et al. did not find a decrease in gapping when tightening a Krackow stitch [20]. For these reasons, we investigated the simple and cross-lock Bunnell repairs because the suture design is more suitable for pretension, even though the use of a simple, rather than locking, configuration of the Bunnell repair is itself a risk factor for gapping [25].

The biomechanical properties of our porcine tendon tension-free Bunnell repair are similar to the results of other biomechanical studies on human test specimens [5, 13]. For example, Gebauer et al. reported a maximum tensile strength of 139 ± 29.8 N after Bunnell repair using a size 1 PDS, which is close to our measurement of 145 ± 27.9N [5]. While the tensioned cross-lock Bunnell assessed here showed a maximum force of 192.8 ± 29.5 N, other repair techniques can reach significantly higher repair strengths. Ortiz et al. examined multiple strand repairs and the use of FibreWire™ for Achilles tendon repair, reporting a maximum strength of up to 675 N [4]. Nevertheless, compared with other two-strand repair techniques, the cross-lock Bunnell repair showed an improved tensile strength [5]. According to Orishimo et al., sutured Achilles tendons have to bear tension loads between 20 and 100 N under passive ankle motion without forced dorsiflexion [26]. The pretensioned suture techniques investigated here would probably allow such a postoperative treatment protocol, but early walking in a walking boot cannot be recommended because the repair in that situation has to bear loads of 190 to 369 N [27, 28].

One clear limitation of this ex vivo porcine model should be acknowledged, which is the tendon was transected by a clear cut, whereas in a typical tendon rupture, the ends are frayed and gap formation may be different.

## Conclusion

Suture pretension may prevent gapping and lengthening after surgical tendon repair. The simple and cross-lock Bunnell sutures are both suitable for tensioning, but the cross-lock Bunnell demonstrates better repair strength. Therefore, the tensioned cross-lock Bunnell may be an effective suture technique for clinical use.
